# Comparative effects of *Dracocephalum moldavica* L. and probiotic on performance and health parameters of broiler chickens challenged by dexamethasone-induced stress

**DOI:** 10.5713/ab.24.0698

**Published:** 2025-02-27

**Authors:** Seyed Heidar Hayatolgheibi, Mahdi Hedayati, Saeed Khalaji, Hossein Rajaei-Sharifabadi, Ahmad Reza Seradj

**Affiliations:** 1 Department of Animal Science, Faculty of Agriculture, Malayer University, Malayer, Iran; 2 Departament de Ciència Animal, Universitat de Lleida, Lleida Spain; 3 Agrotecnio CERCA Center, Lleida, Spain

**Keywords:** Cecal Microbiota, Dexamethasone Challenge, Immune Response, Intestine Histology, Lactobacillus-based Probiotic, Medicinal Plants

## Abstract

**Objective:**

Environmental stressors negatively affect poultry productivity by increasing oxidative stress levels. This study evaluated the performance and health status of broilers supplemented with either the medicinal plant *Dracocephalum moldavica* L. powder (DP) or a commercial probiotic (Lactofeed) in the context of oxidative stress induced by dexamethasone.

**Methods:**

A total of 300 one-day-old chicks were enrolled in a completely randomized design with a factorial arrangement of six treatments, in each consisting five replicates of 10 chicks. The experimental treatments were: 1) basal diet without feed additives and no challenge, 2) basal diet supplemented with 0.01% Lactofeed, 3) basal diet with 0.4% DP, 4) basal diet and challenged with dexamethasone (2 mg/kg body weight), 5) basal diet with 0.01% Lactofeed and challenged with dexamethasone, and 6) basal diet with 0.4% DP and challenged with dexamethasone. Growth performance, carcass traits, and internal organ weights were evaluated. Gastrointestinal pH and ileum histology were assessed on days 28 and 42, while cecal microbial counts were determined on days 14, 28, and 42. Serum samples collected on days 14, 28, and 42 analyzed for antibody titers against Newcastle disease and avian influenza viruses, along with white blood cell counts.

**Results:**

The dexamethasone challenge negatively impacted performance, certain carcass traits, white blood cell count, and ileum villus height (p = 0.039), while it reduced cecal Escherichia coli and coliform counts (p = 0.001). It suppressed humoral immune response to the Newcastle disease virus on day 28, whereas on day 42, it enhanced the response. Lactofeed and DP additives significantly improved feed intake, body weight gain, white blood cell count, intestinal histology, and microbiota, largely irrespective of dexamethasone challenge.

**Conclusion:**

While dexamethasone challenge induced various physiological stresses in broilers, both Lactofeed and DP feed additives demonstrated potential in mitigating these effects, improving overall performance and gut health parameters regardless of stress conditions.

## INTRODUCTION

Broiler chickens are highly susceptible to a variety of stressors, both environmental and physiological in nature. These stressors can have far-reaching consequences, negatively impacting feed consumption, nutrient digestion, and amino acid utilization. As a result, performance and productivity of broiler chickens are often compromised, potentially leading to detrimental effects on health and welfare [1]. Moreover, stress-induced changes can also deteriorate meat quality, further compounding the challenges faced by the poultry industry [2]. The underlying mechanisms by which stress exerts these deleterious effects involve the activation of the hypothalamic-pituitary-adrenal axis, leading to the release of glucocorticoid hormones like corticosterone. These hormones play a crucial role in regulating homeostatic processes, including gluconeogenesis, immune function, and inflammatory responses [3].

Dexamethasone, a synthetic glucocorticoid, has been commonly used as a pharmacological tool to induce physiological stress in avian species, as it has a high affinity for glucocorticoid receptors and can effectively mimic the adverse effects of elevated endogenous corticosteroids [4,5]. By employing dexamethasone-induced stress models, researchers have been able to investigate the complex interplay between stress, nutrient metabolism, and overall performance of livestock animals. This approach has provided valuable insights into the physiological mechanisms underlying the deleterious consequences of stress in poultry, paving the way for the development of targeted interventions and management strategies to enhance the resilience and productivity of commercial broiler flocks [5–7].

The use of medicinal plants and phytochemicals as non-hazardous feed additives for poultry has gained considerable attention in recent decades. These natural compounds, such as phenolic compounds and essential oils from aromatic plants, have demonstrated antimicrobial properties and the potential to modulate the immune system and gastrointestinal microbiome of poultry [8,9]. By improving the resistance of chickens to various stressors and enhancing their antioxidant capacity, these phytogenic additives have been shown to mitigate the detrimental effects of stress on poultry performance and productivity. One group of phytochemicals that has garnered particular interest is the aromatic herbs from the *Lamiaceae* family, such as *Dracocephalum moldavica* L., also known as Melissa officinalis or lemon balm. *Dracocephalum moldavica* L. contains a diverse array of bioactive compounds, including monoterpenoid aldehydes, flavonoids, and volatile oils like geranium, linalool, citral, and citronellal, which contribute to its antispasmodic, antioxidant, antibacterial, antiviral, anti-inflammatory, and antihypertensive properties [10,11].

In addition to the use of phytogenic additives, the incorporation of probiotic microorganisms, such as *Lactobacillus* species, into poultry diets has emerged as a promising strategy to support gut health, modulate the immune system, and mitigate the negative impacts of stress [12]. Probiotics have been shown to competitively exclude pathogenic bacteria, produce antimicrobial compounds, and enhance the intestinal barrier function, thereby improving the overall health and performance of poultry under both normal and stressful conditions [13,14].

While the individual benefits of probiotics and phytogenic feed additives have been explored in poultry production, limited information is available on the comparative efficacy of these two approaches under conditions of dexamethasone-induced oxidative stress. Therefore, the present study aimed to comparatively evaluate the effects of a *Lactobacillus*-based probiotic (Lactofeed) and *Dracocephalum moldavica* L. powder (DP) on the performance, health, and immune response of broiler chickens challenged by dexamethasone-induced oxidative stress.

## MATERIALS AND METHODS

### Animal care

All animals used in this study were cared for in accordance with the guidelines of the Iranian Council of Animal Care (NO. IACUC95). The study’s protocol was reviewed and approved by the Animal Care and Use committee, Department of Animal Science, Malayer University, Malayer, Iran (89/4-3-535, Oct. 27/2019).

### Birds and experimental treatments

In this experiment, 300 one-day-old male broiler chicks (Ross 308) were randomly assigned to 6 experimental groups, each consisting of 5 replicates (pens) with 10 chicks per pen. The experiment was conducted over a 42-day period. Three corn-soybean meal-based diets were formulated to meet all nutrient requirements for the three broiler growth phases (Table 1). The experimental treatments were as follows: 1) basal diet without feed additives and no challenge, 2) basal diet with 0.01% Lactofeed, 3) basal diet with 0.4% of DP, 4) basal diet challenged with dexamethasone, 5) basal diet with 0.01% of Lactofeed and challenged with dexamethasone, and 6) basal diet with 0.4% of DP and challenged with dexamethasone. Dexamethasone (Dexacoid; Nasr Fariman Co., Torbat-e Jam, Iran) was administered to the challenged groups at a dose of 2 mg/kg body weight. The challenge was performed on two consecutive three-day periods (days 25 to 27 and days 39 to 41) via intramuscular injection into the right chest muscle of the chickens. A commercial probiotic (Lactofeed; Takgene Co., Tehran, Iran), containing a variety of naturally occurring gastrointestinal microorganisms that produce lactic acid, was used in this study. The active ingredients were *Lactobacillus acidophilus* and *Lactobacillus casei*, at a concentration of 1×10^8^ CFU/g. DP was freshly prepared from summer crops in Malayer, Iran. The plants were shade-dried with free air flow after removing excess parts. The dried material was then ground using an electric mill. The resulting powder was stored at room temperature until use.

### Growth performance

Feed intake (FI) and body weight were recorded weekly using a digital scale with an accuracy of 0.01 g. Feed was weighed before being provided, and any refusals were collected, weighed, and recorded to determine the FI. Body weight was measured at the same time each week to minimize variations due to gut fill. Feed conversion ratio (FCR) was calculated as FI divided by body weight gain (BWG).

### Blood sampling and analysis

At 2, 4, and 6 weeks of age, brachial vein blood samples were collected from two birds in each pen into non-heparinized tubes and centrifuged at 3,000×g for 10 min to separate serum. The serum samples were stored at −20°C for further analysis. Antibody titers against Newcastle disease (ND) and Avian Influenza (AI) viruses were determined by the hemagglutination inhibition test. Differential blood cell count was determined in a hematology laboratory using a hemocytometer slide (Neuobauer®) as described by Hedayati and Manafi [15].

### Microbial analysis of cecal contents

On day 14, 28, and 42 of the experiment, two birds from each pen were randomly selected and euthanized by cervical vertebra displacement for microbial study. One gram of cecal content was collected using sterile forceps and transferred to a sterile sample container. All samples were transferred to the laboratory at 4°C under completely sterile conditions. Changes in the microbial population were evaluated in sterile phosphate-buffered saline (PBS, pH 7.2) by serial dilution. One mL of each sample was used on the following culture media: Salmonella-Shigella agar, MacConkey agar, and Eosin Methylene Blue agar, which had been previously prepared for the growth of coliforms, Salmonella, and *Escherichia coli* bacteria. The samples were spread across the entire culture medium using a sterile swab. The culture was performed under a biosafety hood in the presence of a flame. Finally, bacteria were incubated at 37°C for 24 hours [15].

### Ileal histology assay

For the ileal histology assay, the digestive tract was removed from two randomly selected birds per pen, which were euthanized by cervical vertebra displacement on days 28 and 42. The ileum was separated from Meckel’s diverticulum up to 1 cm proximal to the ileocecal junction and then dried with desiccant paper. A 5 cm section was taken from the middle of the ileum and gently flushed with PBS (pH 7.2). The ileum samples were fixed in 10% formalin. Tissue sections (2 μm) were cut using a microtome (Leitz-1512 Microtome; Leitz, Wetzlar, Germany), floated onto slides, and stained with hematoxylin and eosin. To measure villus height and crypt depth, images from samples were taken using a light microscope equipped with a digital camera. Twelve images from 4 tissue sections were captured, and 24 villus heights and crypt depths were measured using imaging software (ToupView 7.1), as described by Hedayati and Manafi [15].

### Gastrointestinal pH measurement

Digesta samples were collected from the proventriculus, gizzard, and duodenum of two birds per pen, which were euthanized on days 28 and 42. The samples were collected immediately after euthanasia. The pH of the digesta was measured using a calibrated digital pH meter (HI 98107 pHep pH Meter; Hanna Instruments, Cluj Napoca, Romania).

### Relative weights of carcass and internal organs

Chickens euthanized on day 42 of the experiment underwent a comprehensive carcass and organ assessment. The carcass was prepared by removing the skin to expose the internal organs. The weights of the liver, spleen, heart, gizzard, thymus, as well as the eviscerated carcass, breast, and thigh, were measured using a digital pan balance with an accuracy of 0.001 g. To standardize comparisons across experimental groups and account for individual bird size variations, all measurements were expressed as grams per 100 g of body weight.

### Statistical analysis

All statistical analyses were performed using SAS (version 9.2; SAS Institute Inc., Cary, NC, USA) software [16]. The experiment was designed as a completely randomized design with a 2×3 factorial arrangement, consisting of two challenge conditions (with or without dexamethasone) and three dietary treatments (control, probiotic, and herbal supplement). Data were analyzed using the PROC MIXED procedure of SAS. The statistical model included the fixed effects of dexamethasone challenge, dietary treatments, and their interactions. For performance parameters (FI, BWG, and FCR), the pen was considered the experimental unit, while individual birds served as the experimental units for other measurements. Least squares means (LSMeans) were calculated for all effects. Pairwise comparisons between LSMeans were performed using the Duncan’s Multiple Range Test. Differences were considered statistically significant at p≤0.05.

## RESULTS

### Performance parameters

The dietary supplementation with Lactofeed and DP under dexamethasone-induced stress significantly influenced broiler FI on days 7 (p = 0.01) and 28 (p = 0.05). Furthermore, a significant interaction between dexamethasone-induced stress and these additives was observed on days 14 (p = 0.05) and 28 (p = 0.03). FI remained unaffected by the experimental diets during other time periods (Table 2). BWG analysis revealed significant differences between dexamethasone-injected and non-injected broilers on days 14, 28, 35, and 42 (Table 3). Broilers receiving Lactofeed and DP exhibited significant BWG differences on days 7 (p = 0.037) and 28 (p = 0.026), with the Lactofeed group demonstrating the highest BWG. However, no significant interaction was observed between dexamethasone-induced stress and the additives regarding BWG (p>0.05). Table 4 presents the results of FCR in broiler chickens. FCR was significantly affected in by dexamethasone administration on days 14 (p = 0.012), 28 (p = 0.009), 35 (p = 0.002), and 42 (p = 0.005), with higher FCR observed in the dexamethasone-challenged groups compared to unchallenged groups. Neither the addition of Lactofeed nor DP had a significant effect on FCR. Additionally, no interaction between the dexamethasone challenge and feed additives was observed for FCR (p>0.05).

### Carcass traits and relative organ weight

At 42 days of age, significant differences were observed in the relative weights of carcass (p = 0.001), thigh (p = 0.022), liver (p = 0.001), spleen (p = 0.001), and thymus (p = 0.026) between dexamethasone-challenged chickens and those non-challenged. The main effect of feed additive on the relative weight of organs was non-significant (Table 5). There were interactions between dexamethasone-induced stress and feed additives for the relative weights of breast (p = 0.026) and heart (p = 0.002).

### Gastrointestinal pH

The effect of experimental treatments on the pH of proventriculus, gizzard, and duodenum digesta at days 28 and 42 of age is presented in Table 6. The administration of dexamethasone increased proventriculus digesta pH on day 28 compared to non-challenged chickens (p = 0.033). The main effect of the feed additives as well as their interaction with dexamethasone challenge on the pH of gastrointestinal tract were non-significant (p>0.05).

### Immune parameters and white blood cell counts

As shown in Table 7, dexamethasone administration significantly affected antibody titers against ND virus on days 28 (p = 0.006) and 42 (p = 0.001) of age. While dexamethasone initially reduced ND antibody titers on day 28, chickens in challenged groups exhibited higher titers on day 42. Both Lactofeed and DP significantly influenced ND antibody titers on day 28. On day 42, DP increased ND antibody titers (p = 0.021), whereas Lactofeed-fed chickens showed lower antibody titers against AI virus compared to those receiving no feed additives (p = 0.002). Furthermore, significant interactions between dexamethasone administration and feed additives were observed for ND antibody titers ondays 14 (p = 0.002), 28 (p = 0.001), and 42 (p = 0.001).

Dexamethasone-induced stress significantly affected the counts of heterophil (p = 0.014) on day 14, monocytes (p = 0.034) and eosinophils (p = 0.009) on day 28, and heterophils (p = 0.005) and basophils (p = 0.001) on day 42. Lactofeed and DP supplementation significantly influenced the counts of heterophils (p = 0.001) and eosinophils (p = 0.017) on day 28, as well as the count of lymphocytes on day 42 (p = 0.016). The interactions between dexamethasone-induced stress and the feed additives were significant for heterophils (p = 0.001), lymphocytes (p = 0.051) and eosinophils (p = 0.019) on day 28, and heterophils (p = 0.012) and lymphocytes (p = 0.022) on day 42. Other parameters were not significantly affected by the treatments (Tables 8–10).

### Ileal histology and cecal microbiota

On day 28 of age, no significant effects of the experimental treatments on ileal histology were observed. However, on day 42 of age, dexamethasone administration significantly reduced ileum villus height (p = 0.039) in challenged chickens. Lactofeed and DP had significant positive effects on ileum villus height (p = 0.015). Additionally, crypt depth was significantly higher in DP-supplemented chickens compared to those receiving Lactofeed (p = 0.051). No interactions between dexamethasone-induced stress and feed additives were observed for ileal histology parameters (Table 11).

The microbial counts in intestinal digesta are presented in Table 12. Dexamethasone-induced stress significantly affected Salmonella spp. counts at 14 days of age, and coliform and *Escherichia coli* (*E. coli*) counts on days 28 and 42 (p = 0.001). DP supplementation significantly reduced *E. coli* populations at 28 days of age (p = 0.054), while Lactofeed supplementation significantly reduced *E. coli* at 42 days (p = 0.051). The interaction between dexamethasone-induced stress and the additives significantly affected coliform counts on day 28 (p = 0.054) and Salmonella counts on day 42 (p = 0.028).

## DISCUSSION

Various forms of stress have been shown to directly or indirectly activate the hypothalamic-pituitary-adrenal axis, leading to the release of corticosterone into the bloodstream. Glucocorticoids secreted in response to stress trigger proteolytic mechanisms in muscles, especially skeletal muscles, causing muscle damage. This stress response also weakens the immune system, particularly affecting lymphoid tissues [17]. In broilers, corticosterone impacts the growth, and feed consumption control mechanisms, resulting in reduced feed efficiency [18]. Recent research has demonstrated that injecting corticosterone into broilers for seven days reduces insulin-like growth factor type 1 and impairs thyroid hormone metabolism, consequently reducing growth rate and FI during corticosterone-induced stress [19]. In the present study, dexamethasone injection resulted in reduced FI and BWG, along with increased FCR, indicating successful induction of physiological stress in the experimental birds. These findings are consistent with Lin et al [20], who induced stress in broilers by adding 30 mg/kg corticosterone to the diet for 12 days, reporting decreased body weight and increased FCR due to corticosterone consumption. Similarly, weight loss has been observed with injections of 0.1, 1.5, and 5 mg dexamethasone per kg body weight in broilers [7]. Interestingly, Song et al [21] reported that injecting chickens with dexamethasone for three days starting at 7 days of age increased FI. This contrasting result highlights the complexity of stress responses in poultry and suggests that the effects of glucocorticoids may vary depending on factors such as age, duration of exposure, and dosage.

Several studies have reported the antioxidant effects of medicinal plants and their active ingredients on improving broiler performance. For instance, Hashemipour et al [22] demonstrated that dietary supplementation with thymol and carvacrol, active components of thyme from the mint family, improved the activity of antioxidant enzymes in broilers. Cross et al [23] found that the use of medicinal plants (mint, rosemary, and thyme) and their essential oils increased FI. Additionally, Nobakht et al [24] reported that the inclusion of 2% herbs in broiler diets numerically improved daily weight gain compared to the control group, likely due to the antibacterial and antifungal effects of the plant compounds used in the experimental groups. However, in the present study, no significant effect of DP was observed on the performance of broiler chickens. This finding aligns with Najafi and Torki [25], who reported that FI of broilers fed diets containing herbs was not affected by experimental treatments. Similarly, Hernández et al [26] found that herbs and their extracts, including peppermint leaves, had no significant effect on FI or overall performance. The variability in results regarding FI across different experiments may be attributed to the high number of influencing factors and their interactions.

In contrast to DP, Lactofeed exhibited significant positive effects on the performance of broiler chickens in our study. These beneficial outcomes may be influenced by general traits of probiotics, such as the production of lactic acid, competitive elimination of pathogenic bacteria, and improvement of intestinal mucosa [27,28]. It is noteworthy that the positive effect of Lactofeed was significant under normal conditions but not under dexamethasone-challenged conditions. The discrepancies in results across studies highlight the complex nature of broiler nutrition and the need for further research to elucidate the mechanisms behind the varying effects of herbal supplements and probiotics. Future studies should aim to standardize experimental protocols and consider these variables to better understand the efficacy of such supplements under different conditions, including stress-induced states.

The reduction in weight of broilers by glucocorticoids can be attributed to their proteolytic effect on specific cells, particularly skeletal muscle cells. Glucocorticoids increase the production of free radicals, leading to atrophy induction in muscle fibers [17]. In the present study, while dexamethasone-induced stress had no significant effect on the relative weight of the breast, reductions in the relative weights of carcass and thigh were observed. Interestingly, DP showed differential effects on the relative weight of breast, with a positive effect under normal conditions and a negative effect under challenged conditions. Bahadori et al [29] reported that the relative weight of thigh in chickens supplemented with dill powder was significantly higher than those fed a control diet. However, the mechanism behind the varying effects of DP under different conditions remains unclear and warrants further investigation.

Intestinal villus play a crucial role in the process of digestion and absorption in the small intestine. Li et al [7] demonstrated that the use of dexamethasone in broiler diets reduces the length of villus and level of absorption in intestinal mucosa while increasing the depth of intestinal crypts. They also reported that the decrease in sodium-dependent glucose transporter genes is another mechanism that inhibits glucose uptake from the gut. In our study, dexamethasone administration had a negative effect on villus height. However, DP increased both villus height and crypt depth under both normal and challenged conditions, while Lactofeed showed positive effect on villus height without significant effect on crypt depth.

Previous studies have indicated that medicinal herbs have a positive effect on gut health and morphology. For instance, using 0.5% dill powder supplement increased the total weight of digestive tract organs and improved FCR [29]. Additionally, the positive effects of probiotics on intestinal health index have been reported. Chichlowski et al [30] found that the inclusion of probiotics in poultry diets increased villus length and width, muscle layer thickness, and the number of goblet cells compared to the control group. Pelicano et al [31] reported that the use of probiotics increased the length of the duodenum in broiler chickens. Furthermore, Rahimi and Khaksefidi [32] observed an increase in the number of lactic acid bacteria spp. in the gastrointestinal tract due to supplementation with probiotic compounds. However, in contrast to these findings and in agreement with our results, some studies [32] concluded that probiotic use had no effect on the histology of ileum and duodenum in broilers at 14 and 42 days of age.

Our study indicated that DP have the potential to increase the antibody titers against ND virus. These findings align with previous research on the immunomodulatory effects of herbal supplements in poultry. Various flavonoid compounds, such as luteolin, ferulic acid, pinene, and umbelliferon, present in dill, possess high antioxidant properties [33]. These compounds have been shown to improve bird performance by enhancing their immune and overall health systems. However, the literature presents mixed results regarding the effects of herbal supplements on immune responses. For instance, Ghalamkari et al [34] reported that the use of Satureja powder had no effect on ND virus antiviral antibody protection. No significant differences were also observed between experimental groups in white and red blood cell counts, percentages of heterophils and lymphocytes, hematocrit, and hemoglobin levels when broilers were fed diets containing different levels of Coriandrum sativum essential oils [35].

Probiotics have also demonstrated immunomodulatory effects in poultry. Rahimi and Khaksefidi [32] reported that probiotic supplementation under heat stress significantly increased the number of white blood cells and decreased the ratio of heterophils to lymphocytes compared to the control group. Fuller [27] suggested that probiotics stimulate the immune system by enhancing antibody production, increasing phagocyte activity, elevating total serum protein levels, and increasing the ratio of globulins to albumin, as well as the number of white blood cells. Despite the positive findings reported in previous studies, the probiotic treatment in the present study showed minimal effects on white blood cell counts, with a notable increase in heterophils, particularly under normal conditions.

The antimicrobial effects of herbal essential oils, particularly on gram-positive bacteria, contribute to their potential benefits in poultry production. Adaszyńska-Skwirzyńska and Szczerbińska [36] explained that this antimicrobial function depends on the lipophilic traits of essential oil molecules, which can penetrate bacterial cell and mitochondrial membranes, causing proton pump collapse and ATP depletion. Ultee et al [37] identified carvacrol as a particularly potent antimicrobial compound, interfering with membrane functions, increasing lipid membrane permeability, and ultimately causing cell death. Burt [38] noted that thyme essential oils with higher phenol concentrations exhibit greater antimicrobial effects. Mohammadi [39] further elaborated on the mechanism of essential oils against pathogenic bacteria, highlighting their hydrophobic properties. These properties allow essential oils to distribute within the lipid sections of the bacterial cell wall, causing structural changes and increased permeability. This leads to the leakage of ions and other vital cellular contents, ultimately resulting in bacterial death.

## CONCLUSION

The dexamethasone treatment clearly elicited a stress response, evidenced by decreased performance, altered carcass traits, and changes in immune parameters and intestinal histology. These effects underscore the significant impact that stress can have on broiler health and productivity. However, the application of both Lactofeed and DP feed additives showed promising results in counteracting these stress-induced changes. Their positive effects on FI, BWG, and various physiological parameters suggest that these additives may enhance the resilience of broilers to stress. Notably, the improvements in intestinal histology and microbiota indicate that these additives may exert their beneficial effects primarily through modulation of gut health. Further research is warranted to elucidate the specific mechanisms by which these feed additives confer their benefits, and to optimize their application in various production scenarios.

## Figures and Tables

**Table 1:** Ingredients and chemical compositions of basal diets

Item	Starter (0–10 d)	Grower (11–24 d)	Finisher (25–42 d)
Ingredient (%)			
Corn	56.70	58.78	63.70
Soybean meal	37.34	34.62	29.32
Soybean oil	1.66	2.78	3.39
Dicalcium phosphate	1.83	1.63	1.46
Limestone	0.87	0.78	0.74
Sodium chloride	0.30	0.30	0.30
Sodium bicarbonate	0.10	0.10	0.10
L-Lysine-HCl (98%)	0.23	0.15	0.16
Dl-Methionine (98%)	0.34	0.28	0.26
l-Threonine	0.13	0.08	0.07
Vitamin and mineral premix^1)^	0.50	0.50	0.50
Calculated chemical composition			
Metabolizable energy (kcal/kg)	2,900	3,000	3,100
Crude protein (%)	22.00	20.80	18.90
Calcium (%)	0.93	0.84	0.78
Available phosphorous (%)	0.46	0.42	0.38
Sodium (%)	0.16	0.16	0.16
Lysine (%)	1.24	1.11	1.00
Methionine (%)	0.63	0.56	0.52
Methionine+cysteine (%)	0.92	0.84	0.78
Threonine (%)	0.83	0.75	0.67
DCAB (mEq/kg)	232	224	200

1) Supplied per kg diet: Vitamin A 9000 IU, vitamin D3 2000 IU, vitamin E 18 IU, vitamin K3 2 mg, riboflavin 6.6 mg, pantothenic acid 10 mg, pyridoxine 3 mg, folic acid 1 mg, thiamin 1.8 mg, B12 15 μg, biotin 0.1 mg, niacin 30 mg, choline 500 mg, selenium 0.2 mg, iodine 1 mg, copper 10 mg, iron 50 mg, zinc 85 mg, manganese 100 mg.

**Table 2:** Effect of Dracocephalum moldavica powder (DP) and probiotic (Lactofeed) on feed intake of broiler chickens under normal or challenged conditions from day 0 to 42 of age (g)

Treatments	0–7 d	0–14 d	0–21 d	0–28 d	0–35 d	0–42 d
Normal	96.04	309.23	690.86	1,146	1,890	3,004
Challenged^1)^	93.64	314.79	684.43	1,145	1,867	2,978
SEM	1.05	2.63	5.20	9.66	22.93	31.42
No feed additives	97.67^a^	307.78	682.76	1,125^b^	1,831	2,931
Lactofeed^2)^	94.77^ab^	315.57	696.24	1,167^a^	1,895	3,002
DP^3)^	92.07^b^	312.70	683.96	1,144^ab^	1,909	3,042
SEM	1.29	3.23	6.37	11.84	28.08	48.38
Normal+no feed additives	93.30	300.77 ^c^	683	1,120^b^	1,797	2,890
Normal+Lactofeed	96.97	319.17 ^a^	708	1,193^a^	1,951	3,077
Normal+DP	97.77	307.77^c^	680	1,125^b^	1,921	3,046
Challenged+no feed additives	90.77	314.79^b^	682	1,130^b^	1,864	2,972
Challenged+Lactofeed	98.37	311.97^b^	683	1,140^b^	1,839	2,926
Challenged+DP	91.77	317.63^a^	687	1,163^ab^	1,897	3,037
SEM	0.87	2.05	3.80	7.80	17.55	23.80
p-values						
Challenge	0.12	0.14	0.39	0.92	0.49	0.56
Feed additives	0.01	0.24	0.27	0.05	0.13	0.14
Challenge×feed additives	0.15	0.05	0.19	0.03	0.10	0.11

1) Intramuscular injection of dexamethasone at 0.3 cc/kg of body weight.

2) Supplement the diet with 0.1 kg of Lactofeed probiotic per ton of feed.

3) Supplement the diet with 4 kg of *Dracocephalum moldavica* powder per ton of feed.

a–c Different superscripts within the same column denote significant differences (p≤0.05).

SEM, standard error of the mean.

**Table 3:** Effect of *Dracocephalum moldavica* powder (DP) and probiotic (Lactofeed) on body weight gain of broiler chickens under normal or challenged conditions from day 0 to 42 of age (g)

Treatments	0–7 d	0–14 d	0–21 d	0–28 d	0–35 d	0–42 d
Normal	125	269^a^	537	751^a^	1,305 ^a^	1,928^a^
Challenged^1)^	122	257^b^	527	715^b^	1,208 ^b^	1,780^b^
SEM	1.21	3.98	5.00	8.98	17.34	26.25
No feed additives	120^b^	255	527	711^b^	1,256	1,867
Lactofeed^2)^	126^a^	271	538	756^a^	1253	1,826
DP^3)^	123^ab^	263	532	732^ab^	1,252	1,870
SEM	1.49	4.87	6.12	10.97	21.24	32.16
Normal+no feed additives	121	255	532	727	1,306	1,935
Normal+Lactofeed	127	281	544	794	1,312	1,914
Normal+DP	127	273	536	733	1,297	1,936
Challenged+no feed additives	120	257	522	695	1,224	1,799
Challenged+Lactofeed	126	262	532	718	1,194	1,738
Challenged+DP	120	254	528	730	1,207	1,804
SEM	1.10	6.90	12.25	21.95	42.48	64.32
p-values						
Challenge	0.125	0.038	0.137	0.008	0.006	0.005
Feed additives	0.037	0.107	0.450	0.026	0.884	0.562
Challenge×feed additives	0.234	0.209	0.975	0.085	0.818	0.863

1) Intramuscular injection of dexamethasone at 0.3 cc/kg of body weight.

2) Supplement the diet with 0.1 kg of Lactofeed probiotic per ton of feed.

3) Supplement the diet with 4 kg of *Dracocephalum moldavica* powder per ton of feed.

a,b Different superscripts within the same column denote significant differences (p≤0.05).

SEM, standard error of the mean.

**Table 4:** Effect of *Dracocephalum moldavica* powder (DP) and probiotic (Lactofeed) on feed conversion ratio of broiler chickens under normal or challenged conditions from day 0 to 42 of age (g/g)

Treatments	0–7 d	0–14 d	0–21 d	0–28 d	0–35 d	0–42 d
Normal	0.767	1.152^a^	1.285	1.527	1.448^b^	1.560^b^
Challenged^1)^	0.765	1.223^b^	1.297	1.603	1.548^a^	1.675^a^
SEM	0.006	0.018	0.008	0.019	0.020	0.020
No feed additives	0.771	1.21	1.296	1.585	1.450	1.574
Lactofeed^2)^	0.766	1.164	1.293	1.565	1.516	1.650
DP^3)^	0.762	1.189	1.285	1.546	1.528	1.628
SEM	0.007	0.022	0.011	0.023	0.024	0.026
Normal+no additives	0.771	1.195	1.286	1.544	1.378	1.495
Normal+Lactofeed	0.764	1.136	1.303	1.503	1.487	1.612
Normal+DP	0.768	1.126	1.268	1.535	1.481	1.574
Challenged+no feed additives	0.754	1.226	1.306	1.626	1.522	1.653
Challenged+Lactofeed	0.778	1.193	1.283	1.589	1.545	1.688
Challenged+DP	0.763	1.252	1.301	1.595	1.576	1.683
SEM	0.015	0.025	0.021	0.046	0.049	0.050
p-values						
Challenge	0.796	0.012	0.380	0.009	0.002	0.005
Feed additives	0.726	0.373	0.771	0.504	0.077	0.103
Challenge×feed additives	0.367	0.331	0.232	0.914	0.481	0.511

1) Intramuscular injection of dexamethasone at 0.3 cc/kg of body weight.

2) Supplement the diet with 0.1 kg of Lactofeed probiotic per ton of feed.

3) Supplement the diet with 4 kg of *Dracocephalum moldavica* powder per ton of feed.

a,b Different superscripts within the same column denote significant differences (p≤0.05).

SEM, standard error of the mean.

**Table 5:** Effect of *Dracocephalum moldavica* powder (DP) and probiotic (Lactofeed) on carcass traits and internal organs weight of broiler chickens under normal or challenged conditions at 42 days of age (g/100g body weight)

Treatments	Carcass	Tight	Breast	Thymus	Spleen	Gizzard	Heart	Liver
Normal	62.07^a^	27.30^a^	33.03	0.25^b^	0.10^a^	1.96	0.69	2.52^b^
Challenged^1)^	57.15^b^	24.57^b^	34.23	0.30^a^	0.07^b^	1.98	0.65	3.18^a^
SEM	0.57	0.79	0.51	0.01	0.005	0.05	0.02	0.08
No feed additives	59.15	26.52	33.70	0.28	0.07	1.95	0.66	2.86
Lactofeed^2)^	59.28	26.16	33.63	0.28	0.09	2.02	0.68	2.92
DP^3)^	60.39	26.14	33.57	0.26	0.09	1.96	0.66	2.76
SEM	0.70	0.96	0.63	0.01	0.006	0.07	0.03	0.09
Normal+no additives	61.36	24.63	32.49^c^	0.27	0.09	1.87	0.75^a^	2.37
Normal+Lactofeed	62.67	24.34	32.12^c^	0.23	0.11	2.03	0.72^ab^	2.69
Normal+DP	62.17	24.72	34.46^ab^	0.25	0.10	1.99	0.59^c^	2.49
Challenged+no feed additives	56.93	25.36	34.76^ab^	0.29	0.06	2.02	0.57^c^	3.34
Challenged+Lactofeed	55.88	28.68	35.27^a^	0.33	0.06	2.00	0.63^bc^	3.14
Challenged+DP	58.61	27.58	32.68^c^	0.26	0.08	1.92	0.74^a^	3.07
SEM	2.44	1.93	1.27	0.03	0.01	0.14	0.06	0.19
p-values								
Challenge	0.001	0.022	0.113	0.026	0.001	0.826	0.299	0.001
Feed additives	0.406	0.568	0.990	0.340	0.266	0.766	0.872	0.596
Challenge×feed additives	0.268	0.485	0.026	0.140	0.202	0.534	0.002	0.191

1) Intramuscular injection of dexamethasone at 0.3 cc/kg of body weight.

2) Supplement the diet with 0.1 kg of Lactofeed probiotic per ton of feed.

3) Supplement the diet with 4 kg of *Dracocephalum moldavica* powder per ton of feed.

a–c Different superscripts within the same column denote significant differences (p≤0.05).

SEM, standard error of the mean.

**Table 6:** Effect of *Dracocephalum moldavica* powder (DP) and probiotic (Lactofeed) on gastrointestinal pH of broiler chickens under normal or challenged conditions at 42 days of age

Treatments	28 d			42 d		
	
	Proventriculus	Gizzard	Duodenum	Proventriculus	Gizzard	Duodenum
Normal	2.89^b^	4.31	6.14	3.49	3.72	6.42
Challenged^1)^	3.21^a^	4.30	6.27	3.48	3.67	6.09
SEM	0.09	0.08	0.25	0.14	0.08	0.18
No feed additives	2.91	3.90	6.15	3.70	3.56	6.09
Lactofeed^2)^	3.04	4.40	6.19	3.68	3.50	6.26
DP^3)^	3.21	4.62	6.29	3.72	3.41	6.41
SEM	0.12	0.10	0.30	0.08	0.18	0.22
Normal+no additives	2.91	4.13	6.13	3.38	3.68	6.45
Normal+Lactofeed	2.71	4.15	6.13	3.64	3.68	6.42
Normal+DP	3.06	4.65	6.17	3.42	3.81	6.38
Challenged+no feed additives	2.90	3.66	6.17	3.74	3.72	5.73
Challenged+Lactofeed	3.37	4.65	6.44	3.36	3.68	6.38
Challenged+DP	3.35	4.59	6.21	3.38	3.63	6.15
SEM	0.24	0.61	0.21	0.34	0.17	0.36
p-values						
Challenge	0.033	0.979	0.273	0.948	0.622	0.131
Feed additives	0.235	0.258	0.601	0.883	0.954	0.487
Challenge×feed additives	0.163	0.540	0.555	0.559	0.642	0.411

1) Intramuscular injection of dexamethasone at 0.3 cc/kg of body weight.

2) Supplement the diet with 0.1 kg of Lactofeed probiotic per ton of feed.

3) Supplement the diet with 4 kg of *Dracocephalum moldavica* powder per ton of feed.

a,b Different superscripts within the same column denote significant differences (p≤0.05).

SEM, standard error of the mean.

**Table 7:** Effect of Dracocephalum moldavica powder (DP) and probiotic (Lactofeed) on antibody titers in broiler chickens under normal or challenged conditions at 14, 28 and 42 days of age (log_2_)

Treatments	14 d		28 d		42 d	
		
	ND	AI	ND	AI	ND	AI
Normal	2.07	2.40	2.47^a^	2.60	1.40^b^	1.87
Challenged^1)^	1.67	2.00	1.93^b^	2.20	2.53^a^	1.80
SEM	0.16	0.14	0.12	0.18	0.11	0.11
No feed additives	2.10	1.90	2.70^a^	2.10	1.80^b^	1.90^a^
Lactofeed^2)^	1.70	2.20	2.00^b^	2.50	1.80^b^	1.40^b^
DP^3)^	1.80	2.50	1.90^b^	2.60	2.30^a^	2.20^a^
SEM	0.20	0.18	0.15	0.22	0.13	0.14
Normal+no additives	1.60^bc^	2.00	2.40^abc^	2.00	1.00^c^	2.00
Normal+Lactofeed	1.00^c^	2.00	2.20^bc^	2.40	1.00^c^	1.40
Normal+DP	2.40^ab^	2.00	2.80^ab^	2.20	2.20^ab^	2.20
Challenged+no feed additives	2.60^a^	1.80	3.00^a^	2.20	2.60^a^	1.80
Challenged+Lactofeed	2.40^ab^	2.40	1.80^c^	2.60	2.60^a^	1.40
Challenged+DP	1.20^c^	3.00	1.00^d^	3.00	2.40^ab^	2.20
SEM	0.40	0.36	0.31	0.44	0.28	0.28
p-values						
Challenge	0.096	0.071	0.006	0.134	0.001	0.686
Feed additives	0.354	0.078	0.002	0.266	0.021	0.002
Challenge×feed additives	0.002	0.087	0.001	0.556	0.001	0.874

1) Intramuscular injection of dexamethasone at 0.3 cc/kg of body weight.

2) Supplement the diet with 0.1 kg of Lactofeed probiotic per ton of feed.

3) Supplement the diet with 4 kg of *Dracocephalum moldavica* powder per ton of feed.

a–d Different superscripts within the same column denote significant differences (p≤0.05).

ND, Newcastle disease virus; AI, avian influenza disease virus; SEM, standard error of the mean.

**Table 8:** Effect of *Dracocephalum moldavica* powder (DP) and probiotic (Lactofeed) on white blood cell count of broiler chickens under normal or challenged conditions at 14 days of age

Treatments	MON	BAS	EOS	LYM	HET
Normal	1.60	0.73	1.13	62.06	35.53^a^
Challenged^1)^	1.4	0.47	1.00	61.40	34.80^b^
SEM	0.15	0.12	0.07	0.23	0.19
No feed additives	1.40	0.70	1.10	62.00	35.10
Lactofeed^2)^	1.50	0.50	1.10	62.70	35.50
DP^3)^	1.60	0.60	1.00	61.50	34.80
SEM	0.18	0.15	0.09	0.28	0.24
Normal+no additives	1.60	0.40	1.00	61.40	35.40
Normal+Lactofeed	1.40	0.60	1.00	61.40	35.80
Normal+DP	1.80	0.40	1.00	61.40	35.40
Challenged+no feed additives	1.20	1.00	1.20	62.00	34.80
Challenged+Lactofeed	1.60	0.40	1.20	61.60	35.40
Challenged+DP	1.40	0.80	1.00	62.60	34.20
SEM	0.37	0.31	0.16	0.57	0.48
p-values					
Challenge	0.363	0.143	0.170	0.056	0.014
Feed additives	0.754	0.656	0.612	0.478	0.080
Challenge×feed additives	0.437	0.177	0.612	0.478	0.486

1) Intramuscular injection of dexamethasone at 0.3 cc/kg of body weight.

2) Supplement the diet with 0.1 kg of Lactofeed probiotic per ton of feed.

3) Supplement the diet with 4 kg of *Dracocephalum moldavica* powder per ton of feed.

a,b Different superscripts within the same column denote significant differences (p≤0.05).

MON, monocyte cells; BAS, basophils; EOS, eosinophils; LYM, lymphocytes; HET, heterophils; SEM, standard error of the mean.

**Table 9:** Effect of *Dracocephalum moldavica* powder (DP) and probiotic (Lactofeed) on white blood cell count of broiler chickens under normal or challenged conditions at 28 days of age

Treatments	MON	BAS	EOS	LYM	HET
Normal	1.73^a^	0.78	1.40 ^a^	62.06	34.46
Challenged^1)^	1.40^b^	0.73	1.00 ^b^	61.73	34.66
SEM	0.10	0.10	0.07	0.17	0.19
No feed additives	1.70^a^	0.70	1.40^a^	62.40^a^	33.80^b^
Lactofeed^2)^	1.80^a^	0.90	1.20^ab^	60.90^b^	35.20^a^
DP^3)^	1.20^b^	0.80	1.00^b^	62.40^a^	34.70^a^
SEM	0.12	0.12	0.09	0.21	0.23
Normal+no additives	2.00	0.60	1.80^a^	62.60	33.00^c^
Normal+Lactofeed	1.80	1.00	1.40^b^	60.80	35.00^a^
Normal+DP	1.40	1.00	1.00^c^	61.80	35.40^a^
Challenged+no feed additives	1.40	1.00	1.00^c^	62.20	34.60^ab^
Challenged+Lactofeed	1.80	1.00	1.00^c^	61.00	35.40^a^
Challenged+DP	1.00	1.00	1.00^c^	63.00	34.00^b^
SEM	0.25	0.25	0.18	0.43	0.47
p-values					
Challenge	0.034	0.380	0.009	0.201	0.473
Feed additives	0.006	0.556	0.0.17	0.001	0.001
Challenge×feed additives	0.266	0.266	0.019	0.051	0.001

1) Intramuscular injection of dexamethasone at 0.3 cc/kg of body weight.

2) Supplement the diet with 0.1 kg of Lactofeed probiotic per ton of feed.

3) Supplement the diet with 4 kg of *Dracocephalum moldavica* powder per ton of feed.

a–c Different superscripts within the same column denote significant differences (p≤0.05).

MON, monocyte cells; BAS, basophils; EOS, eosinophils; LYM, lymphocytes; HET, heterophils; SEM, standard error of the mean.

**Table 10:** Effect of *Dracocephalum moldavica* powder (DP) and probiotic (Lactofeed) on white blood cell count of broiler chickens under normal or challenged conditions at 42 days of age

Treatments	MON	BAS	EOS	LYM	HET
Normal	1.40	0.27^b^	1.13	61.93	35.66^a^
Challenged^1)^	1.20	0.93^a^	1.13	61.66	34.80^b^
SEM	0.12	0.10	0.09	0.19	0.20
No feed additives	1.30	0.70	1.20	61.60^b^	35.60
Lactofeed^2)^	1.40	0.60	1.10	61.40^b^	35.20
DP^3)^	1.20	0.50	1.10	62.40^a^	34.90
SEM	0.14	0.12	0.11	0.23	0.24
Normal+no additives	1.60	0.40	1.20	61.00^c^	35.80^a^
Normal+Lactofeed	1.40	0.20	1.20	61.80^bc^	35.40^b^
Normal+DP	1.20	0.20	1.00	62.20^ab^	35.80^a^
Challenged+no feed additives	1.00	1.00	1.20	62.20^ab^	34.60^bc^
Challenged+Lactofeed	1.40	0.80	1.00	61.00^c^	35.80^a^
Challenged+DP	1.20	1.00	1.20	62.60^ab^	34.00^c^
SEM	0.29	0.24	0.23	0.47	0.49
p-values					
Challenge	0.250	0.001	0.953	0.341	0.005
Feed additives	0.635	0.522	0.780	0.016	0.157
Challenge×feed additives	0.269	0.802	0.483	0.022	0.012

1) Intramuscular injection of dexamethasone at 0.3 cc/kg of body weight.

2) Supplement the diet with 0.1 kg of Lactofeed probiotic per ton of feed.

3) Supplement the diet with 4 kg of *Dracocephalum moldavica* powder per ton of feed.

a–c Different superscripts within the same column denote significant differences (p≤0.05).

MON, monocyte cells; BAS, basophils; EOS, eosinophils; LYM, lymphocytes; HET, heterophils; SEM, standard error of the mean.

**Table 11:** The Effect of *Dracocephalum moldavica* powder (PD) and probiotic (Lactofeed) on ileal histology of broiler chickens under normal or challenged conditions at 28 and 42 days of age

Treatments	28d				42d			
	
	Gobl	VH (μm)	CD (μm)	VH/CD	Gobl	VH (μm)	CD (μm)	VH/CD
Normal	4.55	515	78.01	6.94	4.70	550^a^	72.02	7.68
Challenged^1)^	5.28	532	75.02	7.11	5.42	447^b^	70.10	6.22
SEM	0.25	55.77	4.99	0.63	0.16	27.94	3.72	0.54
No feed additives	5.12	484	84.62	5.82	5.00	391^b^	69.07^ab^	6.58
Lactofeed^2)^	4.88	552	65.41	8.72	5.28	510^a^	62.91^b^	8.23
DP^3)^	4.75	535	79.51	6.53	4.90	594^a^	81.20^a^	7.06
SEM	0.31	68.30	6.11	0.78	0.21	34.22	4.56	0.66
Normal+no additives	4.80	499	93.24	5.39	4.70	445	74.97	6.00
Normal+Lactofeed	4.60	550	63.80	8.92	4.85	621	65.33	9.76
Normal+DP	4.25	496	76.99	6.51	4.55	584	75.77	7.29
Challenged+no feed additives	5.45	469	76.00	6.25	5.30	336	63.18	5.15
Challenged+Lactofeed	5.15	554	67.01	8.53	5.70	399	60.49	6.70
Challenged+DP	5.25	574	82.04	6.56	5.25	605	86.63	6.82
SEM	0.61	136	12.23	1.56	0.41	68.44	9.12	1.32
p-values								
Challenge	0.086	0.833	0.685	0.721	0.157	0.039	0.727	0.338
Feed additives	0.698	0.774	0.150	0.546	0.425	0.015	0.051	0.777
Challenge×feed additives	0.867	0.855	0.415	0.216	0.196	0.117	0.274	0.950

1) Intramuscular injection of dexamethasone at 0.3 cc/kg of body weight.

2) Supplement the diet with 0.1 kg of Lactofeed probiotic per ton of feed.

3) Supplement the diet with 4 kg of *Dracocephalum moldavica* powder per ton of feed.

a,b Different superscripts within the same column denote significant differences (p≤0.05).

Gobl, goblet cells; VH, villus height; CD, crypt depth; VH/CD, villus height to crypt depth ratio; SEM, standard error of the mean.

**Table 12:** The Effect of *Dracocephalum moldavica* powder (DP) and probiotic (Lactofeed) on cecal microbial count of broiler chickens under normal or challenged conditions at 14, 28 and 42 days of age (log10 cfu/g DM)

Treatments	14d			28d			42d		
		
	*E. coli*	*Salmonella*	*Coliforms*	*E. coli*	*Salmonella*	*Coliforms*	*E. coli*	*Salmonella*	*Coliforms*
Normal	8.33	8.22^a^	8.25	8.35^a^	8.30	8.38^a^	8.35^a^	8.26	8.37^a^
Challenged^1)^	8.37	8.11^b^	8.27	8.20^b^	8.26	8.22^b^	8.19^b^	8.28	8.20^b^
SEM	0.031	0.018	0.015	0.014	0.023	0.013	0.012	0.011	0.017
No feed additives	8.32	8.17	8.26	8.29^a^	8.30	8.32	8.31^a^	8.29	8.30
Lactofeed^2)^	8.35	8.14	8.25	8.27^ab^	8.25	8.29	8.25^b^	8.27	8.29
DP^3)^	8.38	8.17	8.27	8.26^b^	8.30	8.28	8.28^ab^	8.24	8.26
SEM	0.032	0.017	0.019	0.011	0.025	0.014	0.014	0.023	0.017
Normal+no additives	8.35	8.21	8.24	8.36	8.23	8.39^ab^	8.39	8.24^b^	8.37
Normal+Lactofeed	8.34	8.21	8.25	8.36	8.23	8.41^a^	8.32	8.22^b^	8.41
Normal+DP	8.31	8.23	8.27	8.31	8.32	8.35^bc^	8.34	8.31^ab^	8.34
Challenged+no feed additives	8.29	8.13	8.29	8.23	8.36	8.26^c^	8.21	8.35^a^	8.24
Challenged+Lactofeed	8.35	8.09	8.26	8.17	8.26	8.18^d^	8.18	8.25^b^	8.18
Challenged+DP	8.46	8.11	8.28	8.19	8.28	8.20^d^	8.19	8.23^b^	8.18
SEM	0.046	0.034	0.038	0.016	0.035	0.029	0.021	0.033	0.034
p-values									
Challenge	0.339	0.001	0.336	0.001	0.232	0.001	0.001	0.485	0.001
Feed additives	0.340	0.476	0.837	0.054	0.247	0.068	0.051	0.252	0.159
Challenge×feed additives	0.081	0.475	0.735	0.137	0.078	0.054	0.447	0.028	0.129

1) Intramuscular injection of dexamethasone at 0.3 cc/kg of body weight.

2) Supplement the diet with 0.1 kg of Lactofeed probiotic per ton of feed.

3) Supplement the diet with 4 kg of *Dracocephalum moldavica* powder per ton of feed.

a–d Different superscripts within the same column denote significant differences (p≤0.05).

*E. coli*, *Escherichia coli*; SEM, standard error of the mean.
